# Validation and Comparative Assessment of Patient Satisfaction and Quality of Life Questionnaires Among Indonesian Nasal Fracture Patients Post-rhinoplasty

**DOI:** 10.1055/s-0045-1813226

**Published:** 2025-12-09

**Authors:** Fateha P. Hakim, Dini W. Widodo, Trimartani Koento, Mirta H. Reksodiputro, Marsen Isbayuputra, Raden Ayu Anatriera Sumarsono, Mikhael Yosia

**Affiliations:** 1 Department of Otorhinolaryngology-Head and Neck Surgery, Dr. Cipto Mangunkusumo National General Hospital, Faculty of Medicine Universitas Indonesia, Jakarta, Indonesia; 2 Department of Community Medicine, Dr. Cipto Mangunkusumo National General Hospital, Faculty of Medicine Universitas Indonesia, Jakarta, Indonesia

**Keywords:** nasal fracture reconstruction, validity, reliability, quality of life, ROE, FROI-17, NOSE, EQ-5D

## Abstract

**Background:**

Successful nasal reconstruction requires both aesthetic and functional restoration. Evaluating outcomes necessitates validated instruments that capture patient satisfaction, functional improvement, and quality of life, including the Rhinoplasty Outcome Evaluation (ROE) questionnaire, Functional Rhinoplasty Outcome Inventory (FROI-17) questionnaire, Nasal Obstruction Symptom Evaluation (NOSE) scale, and EuroQol 5-Dimension (EQ-5D) questionnaire. This study aims to validate the Indonesian versions of the ROE and FROI-17 questionnaires and to assess the correlation of ROE, FROI-17, and NOSE scores with EQ-5D in patients undergoing nasal fracture reconstruction.

**Materials and Methods:**

A two-phase cross-sectional study was conducted. Phase 1 included 30 outpatients for questionnaire validation. Phase 2 involved 40 patients who underwent nasal fracture reconstruction and completed pre- and postoperative assessments using ROE, FROI-17, NOSE, and EQ-5D.

**Results:**

The Indonesian ROE and FROI-17 demonstrated strong item-level validity (Spearman's *ρ =* 0.522–0.861; *p* < 0.05) and high internal consistency (Cronbach's α > 0.7). All questionnaires showed significant postoperative improvements ( *p* < 0.05), with no significant associations with outcome measures and patient baseline characteristics ( *p* > 0.05). ROE scores showed a moderate positive correlation with EQ-5D at all time points ( *ρ =* 0.464–0.542; *p* < 0.05). FROI-17 and NOSE scores demonstrated moderate negative correlations with EQ-5D, significant only postoperatively (FROI-17: *ρ =* –0.46; NOSE: *ρ =* –0.40; *p* < 0.05).

**Conclusion:**

The Indonesian versions of ROE and FROI-17 are valid and reliable instruments for outcome evaluation after nasal reconstruction. ROE showed the strongest correlation with EQ-5D, supporting its use as a comprehensive measure of postoperative quality of life.

## Introduction

Nasal reconstruction following trauma serves both aesthetic and functional purposes, and its success is closely linked to patient satisfaction. 1 While objective parameters such as anatomical alignment and nasal patency remain essential surgical endpoints, they often fail to reflect the patient's perception of outcome adequacy. Postoperative evaluations commonly reveal a discrepancy between physician-reported outcomes and patient-reported experiences, especially regarding psychosocial domains. 2 This discrepancy underscores the need for outcome measures that incorporate the patient's perspective, particularly in procedures like rhinoplasty, where subjective satisfaction significantly influences the perceived success of surgery.

Rhinoplasty is distinct from many other facial procedures due to its inherently subjective outcome evaluation, in which patients' expectations regarding both appearance and nasal function are highly individualized. 3 Furthermore, these expectations are frequently shaped by psychological and social factors, making the assessment of outcomes particularly complex. In such cases, quality of life (QoL) becomes a central endpoint, integrating physical, emotional, and social health domains into a single evaluative framework. 4 To address these complexities, several patient-reported outcome measures (PROMs) have been developed and validated internationally for use in rhinoplasty patients. The Rhinoplasty Outcome Evaluation (ROE) questionnaire is widely employed to assess postoperative satisfaction across functional and aesthetic domains. The Functional Rhinoplasty Outcome Inventory-17 (FROI-17) questionnaire focuses more on nasal function and its impact on daily life, while the Nasal Obstruction Symptom Evaluation (NOSE) questionnaire offers a targeted assessment of nasal airflow limitations. These instruments, when used together, provide a comprehensive subjective analysis of rhinoplasty outcomes. To evaluate general health-related QoL (HRQoL), the EuroQol-5 Dimension (EQ-5D) instrument is often used as a complementary tool. It measures five key domains—mobility, self-care, usual activities, pain/discomfort, and anxiety/depression—thereby offering a broad assessment of overall patient well-being. In this study, the EQ-5D was selected as a generic HRQoL to act as an external reference standard for the disease-specific questionnaires (ROE, FROI-17, and NOSE). Correlation of these instruments with EQ-5D was undertaken to identify which most accurately reflects overall QoL following nasal fracture reconstruction.

Although these instruments have been extensively validated and implemented in various populations, Indonesian versions of the ROE and FROI-17 have not yet been formally translated, culturally adapted, and psychometrically validated. This represents a significant gap, given the increasing demand for outcome-based evaluation in surgical disciplines and the rising number of nasal reconstruction procedures performed in Indonesian clinical settings. Without validated tools, local researchers and clinicians lack reliable means to assess patient satisfaction and QoL, thereby limiting evidence-based surgical evaluation and audit processes.

This study, therefore, aims to address two core objectives. First, to translate, culturally adapt, and validate the ROE and FROI-17 questionnaires for Indonesian-speaking populations using established cross-cultural adaptation methodology. 5 Second, to evaluate the construct validity and responsiveness of the ROE, FROI-17, and NOSE questionnaires by examining their correlations with EQ-5D scores in patients who have undergone nasal fracture reconstruction. Through this approach, the study seeks to establish a reliable set of PROMs for use in Indonesia and to identify which instruments most accurately reflect changes in HRQoL following nasal reconstruction.

## Materials and Methods

This study employed a cross-sectional design and was conducted in two phases. Phase 1 involved the translation, cross-cultural adaptation, and psychometric validation of the Indonesian versions of the ROE and FROI-17 questionnaires. Phase 2 aimed to evaluate the correlation between PROMs (ROE, FROI-17, and NOSE) and HRQoL, as assessed by the EQ-5D, in patients undergoing nasal fracture reconstruction.

Participants were recruited from the Department of Otorhinolaryngology–Head and Neck Surgery, Dr. Cipto Mangunkusumo National General Hospital, Jakarta, Indonesia. In phase 1, 30 outpatients aged 18 to 65 years (18 women, 12 men) were consecutively enrolled between May and June 2024. Participants completed the ROE and FROI-17 questionnaires, which had been translated and adapted into Indonesian following Beaton et al's standardized methodology for cross-cultural adaptation Table S1 and S2 , available in the online version. 5 In phase 2, 40 patients (12 women, 28 men) who had undergone nasal fracture reconstruction between 2021 and 2024 were included. Eligible participants were aged 18 to 65 years and had completed a minimum postoperative period of 2 months (≥8 weeks) before data collection. Given the variability in individual follow-up intervals, postoperative assessments were stratified into two categories for analysis: <6 months and ≥6 months after surgery. Exclusion criteria included a history of allergic rhinitis, chronic rhinosinusitis, sinonasal malignancy, or severe psychiatric disorders such as neuroticism.

Socio-economic status and education were classified according to nationally recognized criteria. Monthly household income was assessed against the Jakarta provincial minimum wage reported by Indonesian Central Bureau of Statistics for 2020. Participants with a household income below IDR 4.2 million (≈USD 280 at the 2020 exchange rate) were categorized as having a low socio-economic level, whereas those with an income at or above this threshold were categorized as having a high socio-economic level. 6 Educational attainment was defined as “basic education” for completion of primary and/or secondary schooling and “higher education” for completion of a college or university degree.

Preoperative and postoperative administrations of the ROE, FROI-17, NOSE, and EQ-5D questionnaires were completed by all subjects. Ethical approval was obtained from the Ethics Committee of the Faculty of Medicine, Universitas Indonesia, and Dr. Cipto Mangunkusumo National General Hospital. All participants provided written informed consent prior to enrollment. Consent included approval for participation in the study, completion of validated questionnaires, and the use of anonymized data for research and publication purposes. Participants were assured of confidentiality, and all data were handled in accordance with institutional and national research ethics guidelines.

The ROE consists of six questions evaluating aesthetic and functional satisfaction, each scored from 0 (worst) to 4 (best). Total scores are converted to a percentage scale, with higher scores indicating greater satisfaction. 4 7 The FROI-17 is a 17-item instrument that assesses nasal function, general well-being, and self-confidence. Each item is scored from 0 (no complaint) to 5 (worst possible), and the overall score is converted into a percentage, with lower scores reflecting higher satisfaction. 4 8 The NOSE questionnaire includes five items measuring nasal obstruction symptoms, scored from 0 (no problem) to 4 (severe problem), and similarly converted to a percentage score, with lower values indicating better outcomes. 3 9 10 The EQ-5D evaluates five domains: mobility, self-care, usual activities, pain/discomfort, and anxiety/depression, each with three response levels. It also includes a visual analog scale ranging from 0 (worst imaginable health) to 100 (best imaginable health), with final scores expressed as percentages, where higher scores denote better QoL. 11

Statistical analysis was performed using SPSS version 27 (IBM Corp, Armonk, New York). In phase 1, construct validity of the ROE and FROI-17 was tested using Pearson ( *r* ) or Spearman (ρ) correlation coefficients, as appropriate. Internal consistency was evaluated with Cronbach's α, where values ≥0.70 were considered acceptable for reliability. In phase 2, bivariate analyses with Kruskal–Wallis H or Mann-Whitney U-tests were conducted to examine the association between questionnaire scores and patient characteristics. Wilcoxon tests were employed to compare preoperative and postoperative scores. Spearman correlation analyses were also conducted to evaluate the relationship between ROE, FROI-17, and NOSE scores and EQ-5D results in preoperative, postoperative, and delta (change) scores. Correlations were interpreted as negligible (ρ = 0.00–0.09), weak (ρ = 0.10–0.39), moderate (ρ = 0.40–0.69), strong (ρ = 0.70–0.89), and very strong (ρ = 0.90–1.00). 12 The statistical significance was set at 5%.

## Results

### Demographic Characteristic

In phase 1, a cross-sectional descriptive-analytical approach was employed to assess the reliability and validity of the Indonesian versions of the ROE and FROI-17 questionnaires. A total of 30 patients were enrolled using a consecutive sampling method ( Table 1 ). Phase 2 evaluated the correlation between the ROE, FROI-17, and NOSE questionnaires and the EQ-5D in assessing QoL following nasal fracture reconstruction. Forty patients were included in this phase, also recruited through consecutive sampling ( Table 2 ).

**Table 1:** Demographic characteristics of patients in phase 1

Subject characteristics	Total ( *n* = 30)
Sex, *N* (%)	
Male	12 (40.0)
Female	18 (60.0)
Age (years), median (IQR)	35.3 (26.8–46.5)
Education level, *N* (%)	
Basic education	13 (43.3)
Higher education	17 (56.7)
Socio economic level, *N* (%)	
Low	22 (73.3)
High	8 (26.7)

Abbreviation: IQΡ, interquartile range.

Note: Demographic characteristics of participants in phase 1 of the study. Categorical variables are presented as frequencies ( *n* ) and percentages (%), while continuous variables are expressed as median with IQR. Variables include sex, age, educational attainment, and socioeconomic status.

**Table 2:** Demographic characteristics of patients in phase 2

Subject characteristics	Total ( *n* = 40)
Sex, *N* (%)	
Male	28 (70.0)
Female	12 (30.0)
Age (years), median (IQR)	26 (19.3–41.8)
Education level, *N* (%)	
Basic education	24 (60.0)
Higher education	16 (40.0)
Socio-economic level, *N* (%)	
Low	24 (60.0)
High	16 (40.0)
Type of nasal reconstruction, *N* (%)	
Closed reduction	5 (12.5)
Septorhinoplasty	35 (87.5)
Type of graft used in septorhinoplasty, *N* (%)	
Septal cartilage	20 (42.9)
Cartilage concha	9 (25.7)
Rib	11 (31.4)
History of nasal reconstruction surgery, *N* (%)	
Yes	5 (12.5)
No	35 (87.5)
Presence of other facial fractures, *N* (%)	
Yes	13 (32.5)
No	27 (67.5)
Time since postoperative evaluation (months), *N* (%)	
< 6 months	19 (47.5)
> 6 months	21 (52.5)

Abbreviation: IQΡ, interquartile range.

Note: Demographic and clinical characteristics of participants in phase 2 of the study. Categorical variables are presented as frequencies ( *n* ) and percentages (%), while continuous variables are expressed as median with IQR. Variables include sex, age, educational attainment, socioeconomic status, type of nasal reconstruction, graft type used during septorhinoplasty, history of nasal reconstruction surgery, presence of concomitant facial fractures, and time elapsed since postoperative evaluation.

### Phase 1: Validation of the ROE and FROI-17 Questionnaires

The validity assessment using Spearman correlation demonstrated statistically significant results for all items. The ROE questionnaire yielded correlation coefficients ranging from ρ = 0.561 to 0.883, while the FROI-17 ranged from ρ = 0.522 to 0.916. All correlations were significant with *p* <0.05, indicating that each item was valid. Reliability testing showed high internal consistency, with a Cronbach's α of 0.888 for the ROE and 0.952 for the FROI-17, confirming that both questionnaires are reliable instruments ( Table 3 ).

**Table 3:** The validity and reliability test of the Indonesian ROE and FROI-17 questionnaire

FROI-17 ( *n* = 17 items)				ROE ( *n* = 6 items)			
**Item**	ρ	*p* -Value	Cronbach's α	Item	ρ	*p* -Value	Cronbach's α
Q1	0.676	<0.001	0.952	Q1	0.880	<0.001	0.888
Q2	0.750	<0.001		Q2	0.561	0.001	
Q3	0.808	<0.001		Q3	0.854	<0.001	
Q4	0.807	<0.001		Q4	0.784	<0.001	
Q5	0.673	<0.001		Q5	0.883	<0.001	
Q6	0.723	<0.001		Q6	0.861	<0.001	
Q7	0.627	<0.001					
Q8	0.809	<0.001					
Q9	0.820	<0.001					
Q10	0.793	<0.001					
Q11	0.796	<0.001					
Q12	0.733	<0.001					
Q13	0.792	<0.001					
Q14	0.832	<0.001					
Q15	0.847	<0.001					
Q16	0.522	<0.001					
Q17	0.916	<0.001					

Abbreviations: FROI-17, Functional Rhinoplasty Outcome Inventory-17; ROE, Rhinoplasty Outcome Evaluation.

Note: Validity and reliability analysis of the Indonesian versions of the ROE and FROI-17 questionnaires is shown. Item-total correlations were assessed using Spearman's rank correlation coefficient (ρ), while internal consistency was evaluated using Cronbach's α. All correlation coefficients were statistically significant ( *p* < 0.05), demonstrating acceptable item validity. The overall Cronbach's α values indicated a high level of internal consistency for both instruments.

### Phase 2: Comparative Assessment of ROE, FROI-17, and NOSE Questionnaires with EQ-5D Scores

**Fig. 1:**
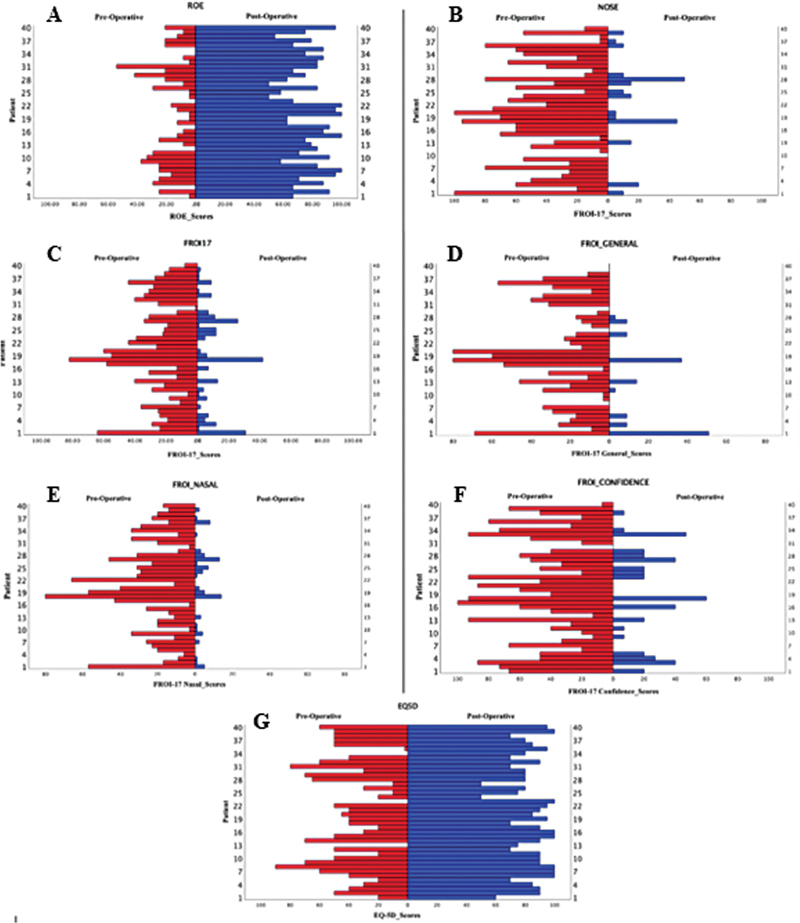
ROE, FROI-17, NOSE, and EQ-5D Questionnaire score distribution before and after surgery. Mirrored horizontal bar plots showing individual preoperative (red bars) and postoperative (blue bars) scores for each patient across seven patient-reported outcome measures: ROE, NOSE, FROI-17 (total and subdomains: General, Nasal, and Confidence), and EQ-5D. Panels **(A)** – **(G)** correspond to ROE (A), NOSE (B), FROI-17 Total (C), FROI-17 General (D), FROI-17 Nasal (E), FROI-17 Confidence (F), and EQ-5D (G). Each plot presents paired distributions for 40 patients ( *y* -axis), with score values shown on the *x* -axis. This format allows visual comparison of individual changes before and after surgery. A general shift from red to blue bars toward more favorable score ranges reflects improvements following either functional or aesthetic nasal surgery. For ROE and EQ-5D, higher postoperative scores indicate better outcomes, while for NOSE and FROI-17, lower postoperative scores indicate clinical improvement. EQ-5D, EuroQol-5 Dimension; FROI-17, Functional Rhinoplasty Outcome Inventory-17; NOSE, Nasal Obstruction Symptom Evaluation; ROE, Rhinoplasty Outcome Evaluation.

The distribution of scores from all questionnaires demonstrated an overall improvement between the preoperative and postoperative assessments ( Fig. 1 ). While the ROE and FROI-17 questionnaires primarily evaluate patient satisfaction, the EQ-5D and NOSE serve as instruments for assessing HRQoL. The greatest mean change (delta) was observed in the ROE questionnaire, with an increase of 62.3%, followed by the EQ-5D (43.9%), NOSE (40.5%), and FROI-17 (23.6%). Within the FROI-17, the self-confidence component exhibited the highest improvement, with a delta score of 39.1%. All comparisons between preoperative and postoperative scores across the questionnaires revealed statistically significant differences ( *p* < 0.05; Table 4 ).

**Table 4:** Comparison of ROE, FROI-17, NOSE, and EQ-5D Questionnaire scores before and after surgery

Questionnaire	Preoperative scores	Postoperative scores	Change (delta) scores	*p* -Value
ROE	15.83 (11.66–20.01)	78.02 (73.34–82.71)	62.29 (56.28–68.10)	<0.001
FROI-17 *(overall score)*	29.53 (24.15–34.91)	5.91 (3.03–8.80)	− 23.62 (−28.07 to −19.17)	<0.001
*Nasal score*	25.35 (19.76–30.94)	6.07 (2.99–9.15)	− 23.23 (−28.32 to −18.13)	<0.001
*General score*	24.85 (17.83–31.87)	3.6 (0.37–6.83)	− 21.25 (−27.32 to −15.18)	<0.001
*Self-confidence score*	50.18 (41.39–58.96)	11.05 (5.97–16.13)	− 39.13 (−47.08 to −31.17)	<0.001
NOSE	46.13 (37.03–55.22)	5.63 (2.04–9.21)	− 40.5 (−48.99 to −32.01)	<0.001
EQ-5D	40.05 (32.81–47.29)	83.88 (79.61–88.14)	43.83 (36.47–51.18)	<0.001

Abbreviations: EQ-5D, Euro Quality of Life 5-Dimension; FROI-17, Functional Rhinoplasty Outcome Inventory-17; NOSE, Nasal Obstruction Symptom Evaluation; ROE, Rhinoplasty Outcome Evaluation.

Note: Comparison of ROE, FROI-17, NOSE, and EQ-5D Questionnaire scores before and after surgery of participants in phase 2 of the study. Variables are presented as mean (minimum–maximum scores). Correlations were assessed using the Wilcoxon test. All correlation coefficients were statistically significant ( *p* < 0.05).

Evaluation of the EQ-5D domain scores also showed a postoperative reduction in the percentage of patients reporting problems across all five dimensions. Among these, a statistically significant improvement was specifically noted in the “usual activities” domain ( *p* < 0.05; Table 5 ). However, no significant associations were found between the changes in questionnaire scores and baseline demographic or clinical characteristics of the subjects ( *p* > 0.05; Table 6 ).

**Table 5:** Correlation of ROE, FROI-17, and NOSE with EQ-5D questionnaires in preoperative, postoperative, and value change assessments

Questionnaire	EQ-5D	*p* -Value
Preoperative		
ROE	0.542	<0.001
FROI-17	−0.297	0.063
NOSE	−0.107	0.512
Postoperative		
ROE	0.511	0.001
FROI-17	−0.455	0.003
NOSE	−0.400	0.011
Change (delta) scores		
ROE	0.464	0.003
FROI-17	−0.211	0.191
NOSE	−0.221	0.171

Abbreviations: EQ-5D, Euro Quality of Life 5-Dimension; FROI-17, Functional Rhinoplasty Outcome Inventory-17; NOSE, Nasal Obstruction Symptom Evaluation; ROE, Rhinoplasty Outcome Evaluation.

Note: Correlation of ROE, FROI-17, and NOSE with EQ-5D questionnaires in preoperative, postoperative, and value change assessments of participants in phase 2 of the study. Variables are presented as the correlation coefficient value (ρ). Correlations were assessed using Spearman's test; statistically significant correlation coefficients are indicated in bold ( *p* < 0.05).

**Table 6:** Relationship of EQ-5D Questionnaire preoperative and postoperative scores

	Mobility				Self-care				Usual activity				Pain/discomfort				Anxiety/depression			
	No problem	Some problem	Severe problem	*p* -Value	No problem	Some problem	Severe problem	*p* -Value	No problem	Some problem	Severe problem	*p* -Value	No problem	Some problem	Severe problem	*p* -Value	No problem	Some problem	Severe problem	No problem
Preoperative	28 (70.0)	10 (25.0)	2 (5.0)	0.160	28 (70.0)	10 (25.0)	2 (5.0)	0.160	7 (17.5)	26 (65.0)	7 (17.5)	0.017	3 (7.5)	23 (57.5)	14 (35.0)	0.181	14 (35.0)	15 (37.5)	11 (27.5)	0.061
Postoperative	39 (97.5)	1 (2.5)	0 (0.0)		39 (97.5)	1 (2.5)	0 (0.0)		35 (87.5)	5 (12.5)	0 (0.0)		30 (75.0)	10 (25.0)	0 (0.0)		33 (82.5)	7 (17.5)	0 (0.0)	

Abbreviations: EQ-5D, Euro Quality of Life 5-Dimension; FROI-17, Functional Rhinoplasty Outcome Inventory-17; NOSE, Nasal Obstruction Symptom Evaluation; ROE, Rhinoplasty Outcome Evaluation.

Note: Comparison of EQ-5D before and after surgery of participants in phase 2 of the study. The EQ-5D assesses five indicators: ability to walk/move, self-care, usual activities, pain/discomfort, and anxiety/depression. Associations were assessed using the Kruskal–Wallis H-test; statistically significant correlation coefficients are indicated with *p* <0.05.

**Table 7:** Comparison of ROE, FROI-17, NOSE, and EQ-5D Questionnaire scores with characteristics of study subjects before and after surgery

	Questionnaire	ROE		FROI-17 overall		FROI-17 nasal		FROI-17 general		FROI-17 self-confidence		NOSE		EQ-5D	
Subject characteristics		Pre-	Post-	Pre-	Post-	Pre-	Post-	Pre-	Post-	Pre-	Post-	Pre-	Post-	Pre-	Post-
Sex	Men	12.5 (0–41.67)	81.25 (50–100)	24.12 (1.18–63.53)	2.35 (0–30.59)	21.5 (3–57)	1.43 (0–22.86)	18.5 (0–80)	0 (0–51)	47 (0–100)	0 (0–47)	55 (5–100)	0 (0–20)	40 (0–90)	85 (50–100)
	Women	18.75 (0–54.17	77.09 (50–100)	32.36 (5.88–82.35)	2.36 (0–42.35)	25.5 (3–80)	2.86 (0–40)	21.5 (3–80)	0 (0–37)	53 (13–93)	3.5 (0–60)	37.5 (0–95)	0 (0–50)	50 (0–80)	90 (50–100)
*p* -Value ^M^		0.171	0.434	0.124	0.522	0.233	0.285	0.289	0.079	0.331	0.419	0.301	0.453	0.440	0.485
Level of education	Basic education	10.42 (0–41.67)	75 (50–95.83)	28.24 (1.18–82.35)	4.12 (0–42.35)	17 (3–80)	0 (0–40)	18.5 (0–80)	0 (0–51)	56.5 (0–100)	3.5 (0–60)	35 (0–100)	0 (0–45)	40 (0–90)	90 (50–100)
	Higher education	18.75 (0–54.17)	83.33 (50–100)	24.71 (12.94–60)	1.18 (0–11.76)	26 (6–66)	0 (0–14.29)	20 (3–80)	0 (0–9)	36.5 (13–73)	0 (0–27)	55 (5–100)	0 (0–50)	47.5 (0–80)	85 (50–1000
*p* -Value ^K^		0.359	0.146	0.814	0.115	0.135	0.113	0.836	0.533	0.030	0.254	0.251	0.115	0.477	0.967
Socio-economic level	Low	12.5 (0–41.67)	75 (50–100)	26.47 (5.88–82.35)	4.12 (0–42.35)	20 (3–80)	2.86 (0–40)	17 (0–80)	0 (0–37)	47 (20–100)	3.5 (0–60)	55 (0–95)	0 (0–50)	40 (0–90)	90 (50–100)
	High	12.5 (0–54.17)	83.33 (50–100)	26.48 (1.18–63.53)	0.59 (0–30.59)	24.5 (3.57)	0 (0–37.14)	24.5 (0–80)	0 (0–51)	37 (0–93)	0 (0–47)	50 (5–100)	0 (0–15)	42.5 (0–80)	85 (50–100)
*p* -Value ^M^		0.550	0.190	0.626	0,089	0.364	0.113	0.443	0.195	0.081	0.343	0.664	0.067	0.435	0.337
Evaluation distance	<6 months	12.5 (0–54.17)	79.17 (50–100)	30.59 (1.18–82.35)	1.18 (0–42.35)	29 (3–80)	2.86 (0–40)	20 (0–80)	0 (0–37)	47 (0–93)	7 (0–60)	55 (5–100)	0 (0–50)	40 (0–80)	80 (50–100)
	>6 months	12.5 (0–41.67)	79.17 (54.17–100)	23.53 (5.88–63.53)	2.35 (0–30.59)	20 (3–57)	2.86 (0–14.29)	20 (0–69)	0 (0–51)	47 (13–100)	7 (0–40)	50 (0–100)	3.1 (0–20)	50 (0–90)	90 (60–100)
*p* -Value ^M^		0.595	0.615	0.132	0.989	0.047	0.272	0.439	0.795	0.634	0.561	0.447	0.156	0.891	0.077
Nose reconstruction	Closed reduction	20.83 (4.17–25)	75 (66.67–95.83)	12.94 (1.18–63.53)	0 (0–30.59)	17 (3–57)	0 (0–14.29)	11 (0–69)	0 (0–51)	13 (0–67)	0 (0–20)	15 (5–100)	0 (0–10)	40 (20–70)	90 (60–100)
	Septorhinoplasty	12.5 (0–54.17)	79.17 (50–100)	28.24 (5.88–82.35)	2.35 (0–42.35)	23 (3–80)	2.86 (0–40)	20 (0–80)	0 (0–37)	47 (13–100)	0 (0–60)	55 (0–100)	0 (0–50)	40 (0–90)	85 (50–100)
*p* -Value ^M^		0.422	0.538	0.106	0,097	0.383	0.142	0.294	0.648	0.014	0.168	0.122	0.297	0.504	0.503
Graft septorhinoplasty	Septum	12.5 (0–54.17)	79.17 (50–100)	30.59 (12.94–82.35)	3.53 (0–42.35)	26 (9–80)	5.71 (0–40)	34 (0–80)	0 (0–37)	40 (13–100)	0 (0–60)	55.5 (5–100)	0 (0–45)	40 (10–80)	80 (50–100)
	Conchae	12.5 (0–33.33)	83.33 (50–100)	22.35 (5.88–43.53)	1.18 (0–11.76)	14 (3–66)	0 (0–22.86)	11 (0–57)	0 (0–9)	47 (20–87)	0 (0–40)	55 (0–80)	0 (0–15)	50 (0–90)	90 (50–100)
	Rib	8.33 (0–29.17)	79.17 (62.5–91.67)	28.24 (17.65–40)	4.71 (0–12.94)	20 (6–34)	2.86 (0–14.29)	20 (0–46)	0 (0–14)	60 (27–93)	20 (0–40)	55 (20–80)	0 (0–50)	40 (0–65)	90 (70–100)
*p* -Value ^K^		0.212	0.667	0.226	0.415	0,079	0.097	0.099	0.286	0.328	0.530	0.594	0.420	0.522	0.472
History of nose reconstruction surgery	New	12.5 (0–54.17)	79.17 (50–100)	25.88 (1.18–82.35)	2.35 (0–42.35)	23 (3–80)	2.86 (0–40)	20 (0–80)	0 (0–51)	47 (0–100)	0 (0–60)	50 (0–100)	0 (0–50)	40 (0–90)	85 (50–100)
	Revision	8.33 (0–29.17)	83.33 (66.67–91.67)	29.41 (12.94–40)	3.53 (0–11.76)	17 (3–34)	2.86 (0–2.86)	26 (3–40)	0 (0–9)	60 (40–87)	7 (0–40)	60 (5–65)	0 (0–20)	40 (20–60)	90 (90–100)
*p* -Value ^M^		0.504	0.526	0.683	0.513	0.152	0.340	0.626	0.313	0.168	0.272	0.604	0.426	0.660	0.106
Facial fractures	Yes	12.5 (0–29.17)	75 (54.17–100)	29.41 (8.24–63.53)	3.53 (0–30.59)	20 (6–66)	0 (0–14.29)	20 (0–69)	0 (0–51)	47 (7–93)	7 (0–40)	40 (5–100)	0 (0–20)	40 (0–90)	90 (60–100)
	No	12.5 (0–54.17)	83.33 (50–100)	25.88 (1.18–82.35)	1.18 (0–42.35)	23 (3–80)	2.86 (0–40)	20 (0–80)	0 (0–37)	47 (0–100)	0 (0–60)	55 (0–100)	0 (0–50)	45 (0–80)	85 (50–100)
*p* -Value ^M^		0,053	0.338	0.631	0.353	0.506	0.292	0.544	0.089	0.383	0.259	0.284	0.555	0.254	0.504

Abbreviations: EQ-5D, Euro Quality of Life 5-Dimension; FROI-17, Functional Rhinoplasty Outcome Inventory-17; NOSE, Nasal Obstruction Symptom Evaluation; ROE, Rhinoplasty Outcome Evaluation.

Note: Comparison of ROE, FROI-17, NOSE, and EQ-5D Questionnaire scores with characteristics of study before and after surgery of participants in phase 2 of the study. Associations were assessed using Mann–Whitney U (M) or Kruskal–Wallis H-tests (K), with statistical significance set at *p* <0.05.

**Fig. 2:**
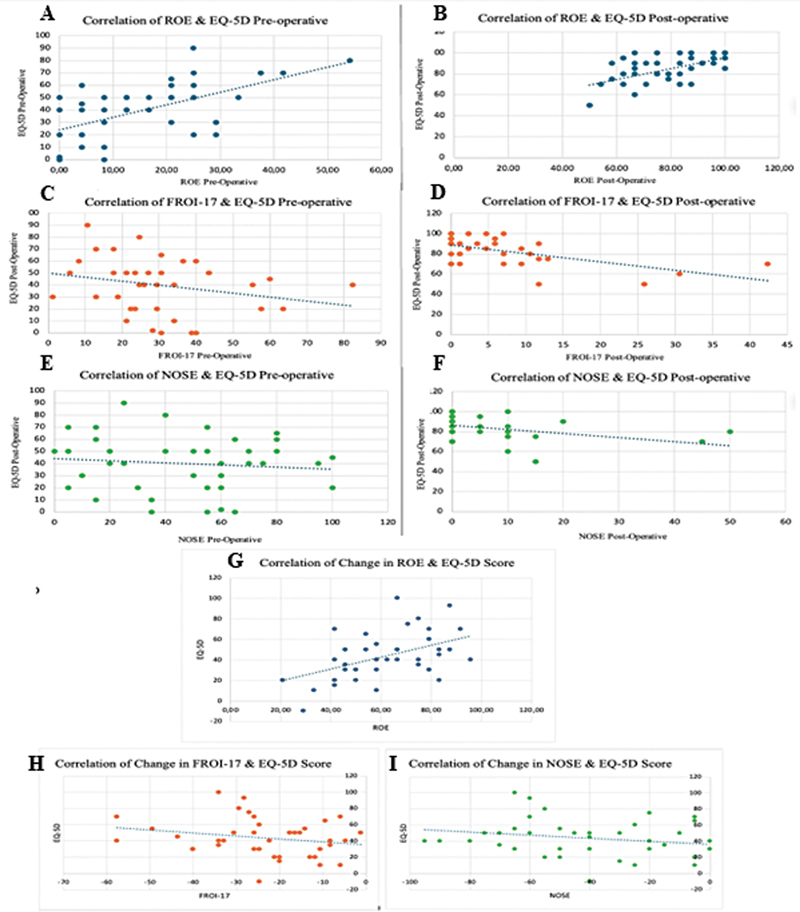
Correlation between changes in patient-reported outcome measures and health-related quality of life (EQ-5D). Panels **(A)** – **(F)** show scatter plots of preoperative and postoperative scores from three patient-reported outcome measures—ROE, FROI-17, and NOSE—in relation to EQ-5D scores. (A) Correlation of preoperative ROE scores with preoperative EQ-5D scores. (B) Correlation of postoperative ROE scores with postoperative EQ-5D scores. (C) Correlation of preoperative FROI-17 scores with preoperative EQ-5D scores. (D) Correlation of postoperative FROI-17 scores with postoperative EQ-5D scores. (E) Correlation of preoperative NOSE scores with preoperative EQ-5D scores. (F) Correlation of postoperative NOSE scores with postoperative EQ-5D scores. Panels **(G)** – **(I)** display the correlation between change scores (delta pre- to post-op) of each questionnaire and the change in EQ-5D score. (G) Correlation of change in ROE score with the change in EQ-5D. (H) Correlation of change in FROI-17 score with the change in EQ-5D. (I) Correlation of change in NOSE score with the change in EQ-5D. Each dot represents an individual patient. Positive correlations indicate that higher satisfaction or better symptom improvement is associated with improved health-related quality of life. Dotted lines represent linear regression trend lines. FROI-17, Functional Rhinoplasty Outcome Inventory–17 (lower scores = greater satisfaction); NOSE, Nasal Obstruction Symptom Evaluation (lower scores = better function); ROE, Rhinoplasty Outcome Evaluation (higher scores = greater satisfaction).

Correlation analysis revealed a moderate positive and statistically significant relationship between the ROE and EQ-5D scores across preoperative, postoperative, and delta scores (ρ = 0.464–0.542, *p* < 0.05). In contrast, both the FROI-17 and NOSE questionnaires demonstrated a moderate negative correlation with EQ-5D, which was statistically significant only in postoperative scores (FROI-17: ρ = –0.455, *p* < 0.05; NOSE: ρ = –0.40, *p* < 0.05). These association are illustrated in Fig. 2 .

## Discussion

The Indonesian versions of the ROE and FROI-17 questionnaires demonstrated strong psychometric properties. Validity testing showed moderate to strong item correlations based on Spearman's coefficient, while internal consistency was excellent, with Cronbach's α values of 0.888 for ROE and 0.952 for FROI-17. These values exceeded those reported in prior validations across other languages, including German, Arabic, Turkish, Persian, and Brazilian adaptations for ROE, 7 13 14 15 16 and even surpassed the original FROI-17's α of 0.880. 8 The observed score changes were also clinically meaningful. Patients with lower preoperative scores showed greater postoperative improvements, suggesting that baseline severity was predictive of delta change. Compared with the normative values proposed by Plath et al, 4 our postoperative ROE and FROI-17 outcomes were superior. Similarly, postoperative improvements in NOSE and EQ-5D exceeded the minimum clinically important differences defined by Fuller et al (≥30 for NOSE; 9.5 for EQ-5D), indicating that rhinoplasty provided satisfactory improvement in nasal obstruction symptoms and overall QoL. 3

Although all PROMs showed significant preoperative-to-postoperative improvement, stratification by demographic or surgical characteristics revealed no significant associations Table 7 ; *p* >0.05. This aligns with the specificity of our cohort, which consisted exclusively of nasal fracture patients. In contrast, previous studies frequently involved heterogeneous populations, including aesthetic-only or functional cases, which may explain divergent findings. 8 17 18 19 20 21 Patients recovering from nasal trauma generally prioritize restoration of pre-injury form and function, while aesthetic patients—often influenced by social media ideals—may develop inflated expectations that predispose them to dissatisfaction or body dysmorphic features. 17 Male predominance in our cohort reflects the demographic reality of higher nasal trauma incidence in men due to occupational and behavioral exposures. Although sex-based differences in satisfaction have been reported, 7 17 our study's sex imbalance limited such comparisons Table 7 ; *p* =0.079-0.485.

Sociodemographic factors such as education and income can influence expectations, especially in aesthetic outcomes. Individuals with higher educational attainment and health literacy often access more information, leading to heightened preoperative expectations. 22 While this may enhance self-efficacy and postoperative confidence, it can also foster unrealistic goals if influenced by distorted standards. 22 23 Similarly, surgical variables—including reconstruction type, graft usage, prior revision, and maxillofacial involvement—did not significantly impact outcomes Table 7 ; most *p* >0.05; EQ-5D preoperative *p* = 0.030. This reflects the individualized surgical planning in our cohort, based on anatomical and radiological evaluation. Preservation rhinoplasty prioritizes maintenance of nasal structures, while structural techniques allow for extensive correction using grafts. 24 Ethnic anatomical differences also guide graft selection, with septal and conchal cartilage commonly used in Caucasian reduction rhinoplasty, and costal cartilage often needed for augmentation in Asian patients. 25

Timing of postoperative evaluation did not significantly affect outcomes in our cohort, whether assessed before or after 6 months. Although studies by Toriumi 26 and Pavri et al 18 suggest edema and contour changes may continue to evolve beyond 3 to 6 months, no time-related difference was observed here Table 7 ; all post operative *p* >0.05. External confounders such as environmental pollutants—especially relevant in urban settings—may affect longer term outcomes and should be considered when interpreting post-rhinoplasty recovery in real-world conditions.

Correlation analyses showed that the ROE questionnaire had the most consistent relationship with the EQ-5D, showing a statistically significant moderate positive correlation across preoperative, postoperative, and delta values. This supports findings by Cingi and Eskiizmir, 19 who reported postoperative improvement in both instruments, though without correlation analysis. ROE's ability to capture both functional and aesthetic satisfaction likely explains its alignment with global HRQoL measures such as EQ-5D. 20 Conversely, FROI-17 showed a statistically significant moderate negative correlation with EQ-5D only in postoperative scores. This contrasts with Bulut et al, 21 who found a significant correlation between FROI-17 and SF-36. The divergence may be due to our cohort's aesthetic priorities, whereas the FROI-17 emphasizes function. The self-confidence subdomain showed the most improvement in FROI-17, reinforcing the relevance of aesthetic recovery in posttraumatic patients. In this context, the brevity of EQ-5D may offer a more focused QoL measure than the broader SF-36.

The NOSE questionnaire also demonstrated a statistically significant moderate negative correlation with EQ-5D postoperatively, but not preoperatively or in delta values. Prior studies have shown that nasal obstruction negatively affects QoL, and that relief of symptoms improves overall health perception. 27 The mean delta NOSE score of 40.5 observed in our study is consistent with results from Rhee et al, 28 further validating the impact of improved nasal patency on patient-reported QoL. Taken together, these findings support the use of multiple PROMs to capture the complex interplay between function, form, and subjective satisfaction in rhinoplasty. Instruments such as ROE, FROI-17, and NOSE should be selected based on preoperative complaint profiles and paired with generic QoL tools like EQ-5D for a comprehensive assessment of outcomes.

This study has several limitations. First, the use of retrospective preoperative self-assessments may introduce recall and reporting bias. Second, the analysis relied solely on patient-reported outcomes without objective functional or anatomical validation. Third, variability in the interval between surgery and postoperative evaluation across subjects may have influenced score changes. Additionally, the absence of long-term follow-up beyond 6 months also precludes evaluation of the durability of patient satisfaction and quality of life improvement. Despite these limitations, the study demonstrated consistent and significant improvements across validated PROMs, supporting the observed correlation between subjective satisfaction and overall QoL. Further high-quality, large-scale prospective studies with a more standardized and longer follow-up period are required to corroborate our findings.

## Conclusion

The Indonesian versions of the ROE and FROI-17 questionnaires are valid, reliable instruments for postoperative assessment in nasal reconstruction. These findings support the ROE as the preferred PROM for integrating aesthetic and functional evaluation in nasal fracture reconstruction. Further prospective studies with a longer follow-up period are warranted to validate our findings.
