# Supplementary material for: Validation and Comparative Assessment of Patient Satisfaction and Quality of Life Questionnaires Among Indonesian Nasal Fracture Patients Post-rhinoplasty
Source: Indian J Plast Surg. 2025 Dec 9;59(2):143–53. doi: 10.1055/s-0045-1813226 (PMC13290373; doi:10.1055/s-0045-1813226)
Supplement: Supplementary file 1 — Supplementary Material [file 10-1055-s-0045-1813226-s2563571.pdf]

Table S1 Indonesian version of ROE

No	ROE
1.	Seberapa suka anda pada bentuk hidung anda?<br>Sama sekali tidak suka (0)<br>Kurang suka (1)<br>Cukup suka (2)<br>Sangat suka (3)<br>Benar-benar suka (4)
2.	Seberapa baik anda dapat bernafas menggunakan hidung anda ?<br>Sangat sulit (0)<br>Sulit (1)<br>Cukup baik (2)<br>Baik (3)<br>Sangat baik (4)
3.	Menurut anda, apakah teman dan orang terdekat anda menyukai hidung anda?<br>Sama sekali tidak suka (0)<br>Agak suka (1)<br>Cukup suka (2)<br>Sangat suka (3)<br>Benar-benar suka (4)
4.	Apakah menurut anda penampilan hidung anda saat ini membatasi aktivitas sosial dan professional anda?<br>Selalu (0)<br>Sering (1)<br>Terkadang (2)<br>Jarang (3)<br>Tidak pernah (4)
5.	Seberapa besar anda meyaini bahwa bentuk hidung anda Adalah bentuk hidung yang paling sesuai/ terbaik dengan wajah anda?<br>Sama sekali tidak yakin (0)<br>Agak yakin (1)<br>Cukup yakin (2)<br>Sangat yakin (3)<br>Benar-benar yakin (4)
6.	Apakah anda ingin mengubah penampilan atau fungsi hidung anda melalui pembedahan?<br>Ya pasti (0)<br>Sangat mungkin (1)<br>Mungkin (2)<br>Mungkin tidak (3)<br>Tidak (4)

Abbreviation: ROE, Rhinoplasty Outcome Evaluation Questionnaire.

Table S2 Indonesian version of FROI-17

No	FROI -17
	Tidak ada masalah<br>Masalah sangat ringan<br>Masalah ringan<br>Masalah sedang<br>Masalah besar<br>Masalah sangat besar
1.	Hidung tersumbat
2.	Hidung meler terus menerus
3.	Lendir mengalir ke tenggorokan
4.	Keluar lendir kental dari hidung
5.	Tenggorokan kering
6.	Terasa ada tekanan pada telinga
7.	Gangguan penciuman
8.	Sulit tidur
9.	Terbangun di malam hari
10.	Mengantuk di siang hari
11.	Sulit berkonsentrasi
12.	Energi menurun
13.	Mudah marah
14.	Tertekan
15.	Kurang percaya diri
16.	Saya malu akan bentuk hidung saya
17.	Keseluruhan dampak buruk dari hidung (bentuk dan fungsinya)

Abbreviation: FROI-17, Functional Rhinoplasty Outcome Inventory questionnaire.

 © 2025. The Author(s).
